# iPSC-derived hindbrain organoids to evaluate escitalopram oxalate treatment responses targeting neuropsychiatric symptoms in Alzheimer’s disease

**DOI:** 10.1038/s41380-024-02629-y

**Published:** 2024-06-05

**Authors:** Cristina Zivko, Ram Sagar, Ariadni Xydia, Alejandro Lopez-Montes, Jacobo Mintzer, Paul B. Rosenberg, David M. Shade, Anton P. Porsteinsson, Constantine G. Lyketsos, Vasiliki Mahairaki

**Affiliations:** 1 Department of Genetic Medicine, Johns Hopkins School of Medicine, 21205 Baltimore, MD USA; 2 The Richman Family Precision Medicine Center of Excellence in Alzheimer’s Disease, Johns Hopkins School of Medicine, 21287 Baltimore, MD USA; 3 Department of Biomedical Engineering, Johns Hopkins School of Medicine, 21205 Baltimore, MD USA; 4 Department of Health Sciences, Medical University of South Carolina, 29425 Charleston, SC USA; 5 Ralph H. Johnson VA Healthcare System, 29401 Charleston, SC USA; 6 Department of Psychiatry and Behavioral Sciences, Johns Hopkins School of Medicine, 21287 Baltimore, MD USA; 7 Department of Epidemiology, Johns Hopkins Bloomberg School of Public Health, 21205 Baltimore, MD USA; 8 Department of Psychiatry, University of Rochester School of Medicine and Dentistry, 14642 Rochester, NY USA; 9 Johns Hopkins Alzheimer’s Disease Research Center, Johns Hopkins School of Medicine, 21205 Baltimore, MD USA

**Keywords:** Diseases, Stem cells

## Abstract

Alzheimer’s disease (AD) is the most common cause of dementia, and the gradual deterioration of brain function eventually leads to death. Almost all AD patients suffer from neuropsychiatric symptoms (NPS), the emergence of which correlates with dysfunctional serotonergic systems. Our aim is to generate hindbrain organoids containing serotonergic neurons using human induced Pluripotent Stem Cells (iPSCs). Work presented here is laying the groundwork for the application of hindbrain organoids to evaluate individual differences in disease progression, NPS development, and pharmacological treatment response. Human peripheral blood mononuclear cells (PBMCs) from healthy volunteers (*n* = 3), an AD patient without NPS (*n* = 1), and AD patients with NPS (*n* = 2) were reprogrammed into iPSCs and subsequently differentiated into hindbrain organoids. The presence of serotonergic neurons was confirmed by quantitative reverse transcription PCR, flow cytometry, immunocytochemistry, and detection of released serotonin (5-HT). We successfully reprogrammed PBMCs into 6 iPSC lines, and subsequently generated hindbrain organoids from 6 individuals to study inter-patient variability using a precision medicine approach. To assess patient-specific treatment effects, organoids were treated with different concentrations of escitalopram oxalate, commonly prescribed for NPS. Changes in 5-HT levels before and after treatment with escitalopram were dose-dependent and variable across patients. Organoids from different people responded differently to the application of escitalopram in vitro. We propose that this 3D platform might be effectively used for drug screening purposes to predict patients with NPS most likely to respond to treatment in vivo and to understand the heterogeneity of treatment responses.

## Introduction

Alzheimer’s disease (AD) is the world’s leading cause of dementia, a gradual process of brain deterioration which manifests through cognitive impairment, loss of function and eventually death [1, 2]. AD is estimated to affect 50–55 million people worldwide, with the number of patients suffering from dementia projected to triple by 2050 [1–3]. A minority of patients (1–2%) present with early onset familial AD, linked to mutations in one of the gene encoding Aβ precursor protein (APP), presenilin 1 and 2 (PSN1 and PSN2) [4]. Most AD cases are sporadic, with a disease onset typically after age 65. There is no available cure for AD at present, and the current landscape for symptomatic treatment is limited in its benefits [5].

The underlying causes of AD are still insufficiently understood, but prominent pathological features include the excessive deposition of extracellular amyloid beta (Aβ) plaques, and abnormal accumulation of hyperphosphorylated tau proteins (neurofibrillary tangles, NFTs) within neurons [6]. The brain degeneration associated with AD is thought to develop over decades after the first pathological features develop in the brain, complicating the possibility of early detection and treatment [4, 7].

Serotonin (5-hydroxytryptamine, 5-HT) is a monoamine neurotransmitter synthesized from L-Tryptophan in a metabolic pathway, which requires the enzymes aromatic amino acid decarboxylase and tryptophan hydroxylase (TPH) [8]. The TPH2 isoform of the latter enzyme is specific to the central nervous system (CNS). 5-HT has a variety of functions in the whole body, but its chemical structure limits it possibility of crossing the blood brain barrier [9]. The 5-HT found in the CNS is locally synthesized in the serotonergic neurons of the raphe-nuclei [10]. Consistent evidence indicates deterioration of the raphe-derived serotonergic system at the earliest stages of AD in at least some individuals [11, 12]. The disruptions in the raphe-nuclei correlate with the nearly universal clinical manifestations of neuropsychiatric symptoms (NPS), which include agitation, depression and psychosis [5, 13]. In clinical settings, selective serotonin reuptake inhibitors (SSRIs) are used effectively to target NPS [14, 15]. However, it is clear that different patients respond differently to these treatments, with as many as 30% not responding at all [16–20]. Better biological predictors of treatment response to individual SSRIs and other therapies are crucial to overcoming this unmet medical need.

Induced pluripotent stem cells (iPSCs) offer a unique in vitro research platform, as they can be differentiated into a variety of brain cell types and region-specialized 3D organoids [21–23]. Crucially, iPSCs can be generated from individuals and serve as models for inter-individual comparisons applying a *Precision Medicine* approach [21, 23]. Using patient-derived iPSCs, organoids can be grown by modeling embryonic development, leading to pre-clinical in vitro models that architecturally mimic physiologically relevant processes of increasing complexity [24–26]. This rapidly growing field can be both directed towards a better understanding of pathological manifestations, and towards improved drug screenings, which would potentially benefit from elements of human heterogeneity being added to in vitro platforms [27–29].

**Fig. 1:**
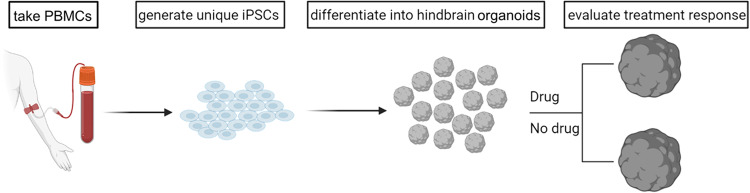
Schematic overview of the project.

Previous studies using 2D iPSC-derived serotonergic neurons, and even 3D organoids containing them, have used Escitalopram in vitro to assess their functionality, e.g., in the context of depressive disorders [30–32]. Our goal is to go a step further and fully explore the individualized medicine approach theoretically offered by iPSC-derived systems, by using samples originating from different people, with a diverse diagnostic background. In this study we aim to reprogram human peripheral blood mononuclear cells (PBMCs) isolated from healthy volunteers (*n* = 3) and AD patients with (*n* = 2) or without (*n* = 1) NPS into iPSCs, and subsequently differentiate them into hindbrain organoids containing serotonergic neurons (5-HT-organoids). The organoids are assessed for potential development of individualized in vitro drug screening platforms, a concept validated by pharmacological treatment with the SSRI escitalopram oxalate [33]. Fig. 1 offers a schematic overview of the workflow.

## Materials and methods

### Reprogramming of PBMCs into iPSCs

iPSC lines were generated as previously described [34, 35]. Briefly, peripheral blood mononuclear cells (PBMCs) were isolated from the blood of 3 healthy individuals as well as from 3 AD patients after obtaining informed consent under the oversight of the Johns Hopkins Institutional Review Board. All samples except for AD_2 and AD_3 were obtained through the Johns Hopkins Alzheimer’s Disease Research Center (ADRC). PBMCs from patients AD_2 and AD_3 are from the ongoing Escitalopram for agitation in Alzheimer’s disease (S-CitAD) clinical trial (NCT03108846) [33]. PBMCs were expanded in culture, enriched for erythroblasts, and subsequently electroporated for the delivery of episomal vectors MOS, MMK and GBX (Addgene) using a 4D–Nucleofector (Lonza) according to the manufacturer’s instructions. After transfection, cells were transferred onto tissue culture plates coated with vitronectin (VTN) in DMEM with 10% FBS (v/v) and supplemented with 5 ng/mL of bone morphogenetic protein4 (BMP4). The following day and thereafter, the medium was replaced with xeno-free and feeder-free Essential 8^TM^ medium (E8, ThermoScientific). Between day 13 and 15 of reprogramming, cells presenting the TRA‐1‐60 pluripotency marker were isolated from the newly generated iPSC colonies using the MACS^TM^ MicroBeads magnetic beads (Miltenyi Biotec). Generated iPSC lines were kept in culture in E8 medium on VTN-coated plates for more than 12 passages before being characterized and used for experiments. For characterization, immunocytochemistry (ICC, see 2.7 below) was performed to check for the presence of multiple pluripotency markers (OCT4, NANOG and TRA-1-60). The iPSC lines underwent flow cytometric analysis to further validate the presence of TRA-1-60 (see 2.4 below).

### Differentiation of iPSCs into hindbrain organoids

Human iPSC lines were differentiated into serotonergic (5-HT) neurons by activating WNT and SHH signaling in a 3D in vitro platform [32, 36, 37]. Briefly, to better mimic brain development, the iPSCs were first used to form embryoid bodies (EBs). Induced PSCs were first centrifuged (200 x g, 1 min) to form aggregates in ultra-low attachment, round-bottom 96-wells-plates (5’000 cells/well, 50 µL/well) in mTeSR^TM^ medium supplemented with the selective ROCK inhibitor y-27632 (Tocris) on day 0. Starting on the following day, the EBs were cultured to differentiate into neural precursors cells (NPCs) specific to the hindbrain over the course of 3 weeks using serotonergic NPC medium (SNm, see Table S1 in the supplementary information for the full composition). On day 1, 50 µL of SNm with double the amount of trophic factors were carefully added to start diluting out the mTeSR. On days 2 and 3, 50 µL of SNm was added to the differentiating EBs. Having reached 200 µL, 50% (100 µL) of SNm medium was exchanged daily until day 21. After the 3 initial weeks, growing NPC organoids were transferred to 6-wells-plates (8 NPC-organoids/well, 2 mL/well), and they were grown in neural differentiation medium (NDm, see Table S1). NDm was exchanged every 3 days. While in the 6-wells-plates, the organoids were kept on an orbital shaker (ThermoFisher, orbital diameter: 22 cm, 50 rpm). Hindbrain organoids containing serotonergic neurons (5-HT-organoids) were ready for characterization and experiments after 6 weeks.

### Organoid area and circularity

In order to evaluate morphological changes of the organoids over time, brightfield images (BF) were taken using an EVOS M5000 microscope (Invitrogen) daily for the first 21 days, then every 3 days until day 42, concurrent with medium changes time points. For the quantification of the area and circularity of the organoids, we developed an in-house algorithm using Python (the full code is available as an open resource on github [38]). Briefly, the images are treated by the code as gray-scale images ranging from 0–255 of intensity values. The organoids are segmented using Felzenswalb algorithm [39] with a previous Gaussian smoothing of the images with a 6 pixels size standard deviation kernel. We enforced a minimum size of 3 pixels for the segmentation. In the next step, to improve the results of the segmentation, we manually set a threshold to differentiate background from organoids to 90 (intensity values). Once the segmentation was performed, the code selects the largest region, excluding background, as a binary mask delimiting the organoid. Finally, the area (A) is then computed integrating the pixels inside the mask. To determine the perimeter (P) of the organoid, we computed the integral of the magnitude of the gradient of the binary mask delimiting the organoid [40]. The circularity (C) or roundness of the organoid can be defined from the area and the perimeter as:

1 $$C=\frac{4\pi A}{{P}^{2}}$$

The more round-like the shape, the closest it can approach the maximum of C = 1, whereas C values smaller than 1 are indicative of non-circular shapes. The values of the area and perimeter are converted from pixel units to mm using a scale bar given by the microscope, the circularity is adimensional. Representative images of segmentation results are found in Figure S1 (supplementary information).

### Flow cytometry

To evaluate the successful reprogramming of PBMCs into iPSCs, cells were dissociated into single‐cell suspensions with TrypLE^TM^ (Life Technologies). They were then washed and resuspended in PBS with 1% BSA (wt/v). They were labeled with the primary antibody anti‐human TRA‐1‐60 (Millipore). For the subsequent detection, iPSCs were labeled with secondary anti‐mouse IgM‐Alexa®Fluor555 (Thermo Scientific) antibody.

To compare iPSCs and the 5-HT-organoids they were differentiated into, iPSCs and 5-HT-organoids were dissociated with TrypLE^TM^ and Gentle cell dissociation reagent (STEM cells technologies) respectively. Cells were washed and resuspended in PBS with 1% BSA (wt/v), following which they were fixed and permeabilized with Cytofix/Cytoperm solution (BD Biosciencence) according to manufacturer’s instructions. Samples were subsequently labeled using AlexaFluor®488 conjugated anti-human TUJ1 and AlexaFluor®647 conjugated anti-human TPH2 antibodies (ThermoScientific). The former is a general neuronal marker, whereas the latter is specific for serotonergic neurons.

All samples were analyzed on a BD LRS Fortessa (BD Biosciences) or on a SH800S cell sorter (SONY Biotechnology). The data was processed using FlowJo^TM^ v10.8.1 software. A full list of the antibodies used for flow cytometry and ICC is available in Table S2 in the SI.

### qRT-PCR

To evaluate the differentiation of iPSCs into 5-HT-organoids, quantitative reverse transcription PCR (qRT-PCR) analysis was performed. Messenger RNA (mRNA) was extracted from cellular pellets of iPSCs and 5-HT-organoids using RNA extraction kit (Zymo research), and it was transcribed into complementary DNA (cDNA) by reverse transcriptase using the Superscript III kit (Invitrogen) following manufacturers’ instructions. The generated cDNAs were used as the template for the qPCR reaction with iTaq Universal SYBR Green (Biorad), which was performed with a CFX Connect thermal cycler (Biorad). The primers used were obtained from Integrated DNA technologies and they were for TRA-1-60 (iPSC marker), NKX2.2 (serotonergic NPC), LMXbI and TUJ1 (neurons), TPH2 and FEV (serotonergic neurons). All forward and reverse primer sequences (purchased from Integrated DNA Technologies) are listed in Table S3 (SI).

### Cryogenic tissue processing and sectioning of the 5-HT-organoids

Hindbrain organoids were washed three times with D-PBS (pH 7.4) and placed in a 1.5 mL centrifugation tube with 1.2 mL of freshly prepared 4% (v/v) paraformaldehyde and left incubating for 18 h at 4 °C. They were then washed for 10 min with D-PBS with 0.1% (v/v) Tween®20 (Sigma) 3 times. For cryoprotection, the organoids were placed in 30% (wt/v) sucrose in D-PBS and left to equilibrate at 4 °C until they did not float in it anymore (ca. 4 h, but it can vary depending on organoid size and density). The organoids were then transferred to an embedding mold which was carefully filled with O.C.T. compound embedding matrix (ThermoFisher). Snap freezing was done by submerging the molds with the embedded organoids in a slurry of dry ice added to 96% ethanol. The frozen organoids were then stored at −80 °C before being sectioned in 10 µm slices at the Johns Hopkins University SOM Microscopy facility.

### Immunocytochemistry (ICC)

Evaluation of pluripotency markers by ICC on adherent human iPSCs was performed as previously described [35]. Briefly, adherent iPSCs in 12-well plates were washed in PBS and fixed with 4% (v/v) paraformaldehyde in PBS (pH 7.4) for 15 min, and permeabilized with Triton X-100 (0.1%, v/v in PBS). To limit non-specific binding, cells were blocked in 10% goat serum (v/v in PBS) for 1 h at 4 °C. They were then stained with either one of the primary antibodies for pluripotency markers, i.e., anti-human TRA-1-60, NANOG and OCT4 at 4 °C overnight. Cells were subsequently washed with PBS, and they were then incubated with the appropriate secondary antibody for 1 h at 4 °C. In the final step, cells were washed with PBS three times, and then stained with DAPI to visualize the nuclei.

Cryo-preserved and sectioned 5-HT-organoids were similarly stained for ICC to confirm the presence of neuronal marker TUJ1, serotonin (5-HT), and neural progenitor cells (NPCs) markers Nestin and NKX2.2 (necessary to determine serotonergic fate) [41]. Confocal fluorescence imaging was performed with a Leica SP8 inverted microscope (DMi8CEL), and the images were analyzed with a Leica LAS X software.

A full list of the antibodies used for flow cytometry and ICC is available in Table S3 in the SI.

### Serotonin (5-HT) measurement and treatment with Escitalopram oxalate

Levels of 5-HT present in the extracellular supernatant were measured by enzyme-linked immunosorbent assay (ELISA) using the Serotonin ELISA kit (Enzo Life Sciences) according to the manufacturer’s instructions. To test the effect of the SSRI escitalopram oxalate, 10 and 100 µM of the drug were added to NDm and incubated with the eight 5-HT organoids for 1 h prior to repeat measurement of supernatant 5-HT. The concentration range was initially chosen based on prior literature [36]; a metabolic activity assay was performed to ensure that the used concentrations were not toxic in our systems (see Figure S3 in the supplementary information).

### Statistical analysis

All experiments were performed in at least 3 biologically independent replicates (n), and at least 3–6 technical repeats (N) unless stated otherwise. The results are presented as mean ± standard deviation (SD). One-way ANOVA test, followed by Tukey’s Honest Significant Difference test, was performed to pairwise evaluate if there were statistically significant mean differences between groups for Fig. 6b–d. The results were displayed using GraphPad Prism version 9.0.0 (121) for Windows, GraphPad Software, San Diego, California USA, www.graphpad.com. Statistically significant results are indicated with their respective p-values and asterisks as follows: p  ≤  0.05 (*), p  ≤  0.01 (**) or p  ≤  0.001 (***).

## Results

### Reprogramming of PBMCs into iPSCs

**Fig. 2:**
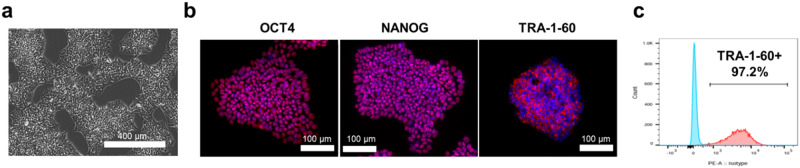
Reprogramming of PBMCs into iPSCs. A representative brightfield microscopy image shows the morphology of newly reprogrammed iPSCs (**a**). Fluorescence microscopy imaging upon staining of iPSCs with pluripotency markers OCT4, NANOG and TRA-1-60 confirms the successful reprogramming (**b**). The enrichment of TRA-1-60 positive cells was additionally validated by flow cytometry (**c**).

Induced PSCs lines were generated using PBMCs originating from 3 healthy individuals and 3 patients with AD, two of whom were also diagnosed with NPS. Homogeneous morphological features were monitored by brightfield (BF) microscopy (Fig. 2a). The presence of pluripotency markers OCT4, NANOG and TRA-1-60 was confirmed by fluorescence microscopy (Fig. 2b). Flow cytometry analysis further validated the successful enrichment of TRA-1-60 positive iPSCs (Fig. 2c).

### Differentiation and characterization of iPSCs into hindbrain organoids

**Fig. 3:**
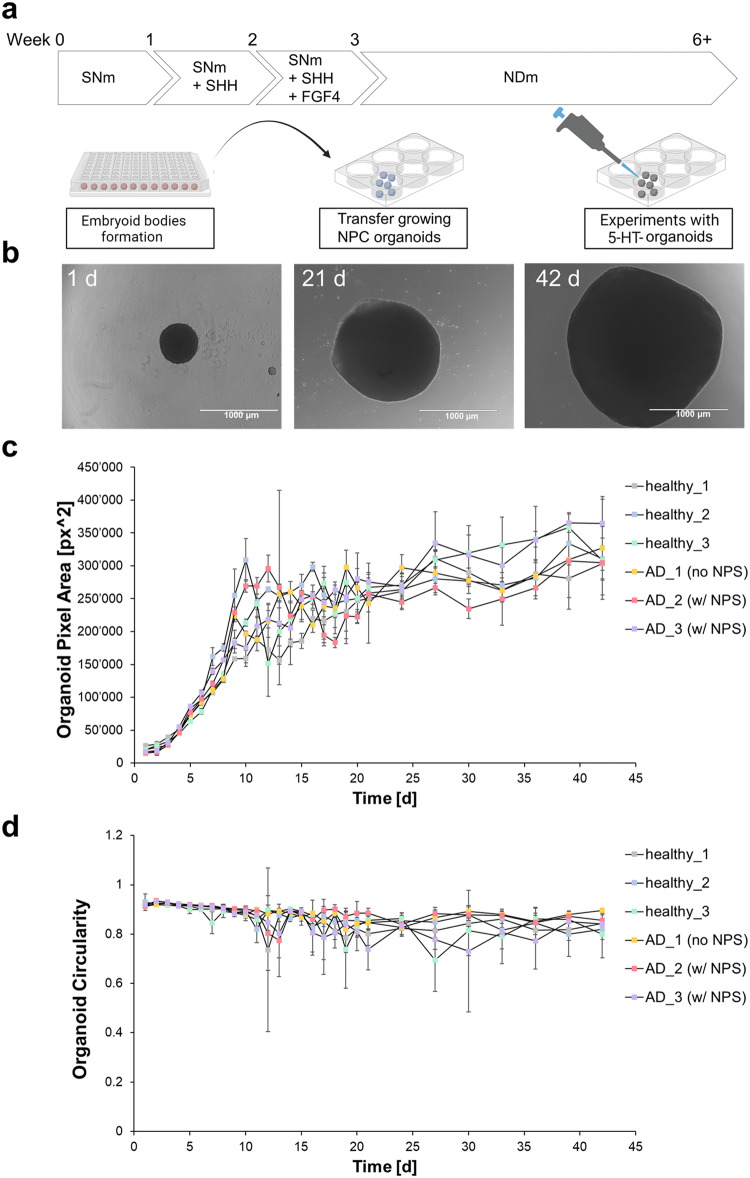
Generation of 5-HT-organoids from iPSCs. Schematic overview of the differentiation protocol (**a**) and representative brightfield (BF) images of the organoids at different stages (1, 21 and 42 d) (**b**). The organoid growth for all 6 cell lines was estimated from their surface area in the BF images (**c**), along with changes in their shape (**d**) (mean ± SD, *n* = 3).

Generation of iPSC-derived 5-HT organoids was successfully accomplished for 6 different cell lines. Figure 3a offers a schematic overview of the differentiation process. BF microscopy images of the organoids at different stages from day 1 to day 42 were used to evaluate their morphology (Fig. 3b). Additionally, the BF area and perimeter were estimated to quantitatively monitor the growth and circularity of the organoids over time (Fig. 3c, d). While general observations about organoid size and shape changes are readily observable by light microscopy, we made an effort to extract and quantify this information from BF images of the organoids with an algorithm we developed in Python (code available on github [38]). The 5-HT-organoids grow continuously over the course of 6 weeks, while having the steepest size increase during the first 10–12 d (Fig. 3c). Their growth rate slows down and almost plateaus for the remainder of the protocol. This could be explained by a biological limit in their growth: once the organoids reach an apparent diameter of around 1 mm, nutrients in the surrounding medium become less and less accessible to cells in the core. The organoids’ size ranges are at their narrowest in the initial stages of differentiation (week 1), and become wider by the end of it, as reflected by their respective standard deviations (SD). Even as the organoids grow, their sizes remain very similar to each other across biologically independent replicates of randomly imaged organoids. Remarkably, growth curves over time, as well as the average organoid size, are very similar across samples originating from iPSC lines derived from the 6 different people. This suggests that the organoids are homogenous and that the protocol is highly reproducible. Measurements of the organoids’ circularity (C, Fig. 3d) indicate and almost perfectly round shape at the beginning of the protocol, with C values above 0.9, and little variation. The roundness slowly decreases over time as the organoids grow and acquire morphological asymmetries. Nevertheless, their circularity still measures consistently between 0.7 and 0.9, regardless of the cell line origin.

**Fig. 4:**
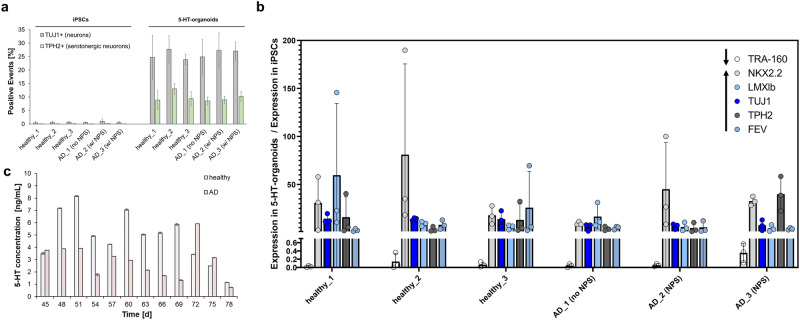
Characterization of 5-HT-organoids. All 6 iPSC lines were successfully differentiated into organoids containing serotonergic neurons as evidenced by the detection of TUJ1 and, more specifically, TPH2 by flow cytometry (**a**) (mean ± SD, *n* = 3). Detailed analysis of different markers by qRT-PCR for all 6 cell lines further validated the differentiation (**b**) (mean ± SD, *n* = 3). The secretion of 5-HT from two representative samples of the organoids was measurable above the lower limit of detection (LOD = 0.49 ng/mL) for over a month after the initial 42 d required for differentiation (**c**) (mean ± SD, *n* = 1).

Flow cytometric detection of TUJ and TPH2 validated the presence of serotonergic neurons in the 5-HT-organoids while being undetectable in the corresponding iPSCs, as shown consistently across all 6 cell lines (Fig. 4a). Figure 4b shows qRT-PCR results for the expression of the relevant hindbrain markers in the 5-HT organoids as compared to the iPSC stage for both healthy and AD cells. Importantly, the pluripotency-marker TRA-1-60 virtually disappeared. Additionally, we could measure the increased expression of neuronal marker TUJ1 (and LMXIb), as well the serotonergic-neurons specific marker TPH2 (along with LMXIb and FEV). The NKX2.2 marker, which is necessary to determine serotonergic neuronal fate in hindbrain development and can be found in serotonergic neurons [36], is still very highly expressed by week 6 at the 5-HT-organoid stage. This suggests that there are remaining NPCs in the organoids and that they are committed to hindbrain fate. The detection of 5-HT in the culture supernatant secreted from the organoids was used to confirm the presence of serotonergic neurons from a fundamental functional perspective. Serotonin levels could be successfully monitored over the course of 36 days after the initial 6 weeks of differentiation (Fig. 4c), thus establishing an effective platform for measuring serotonin release under different conditions.

**Fig. 5:**
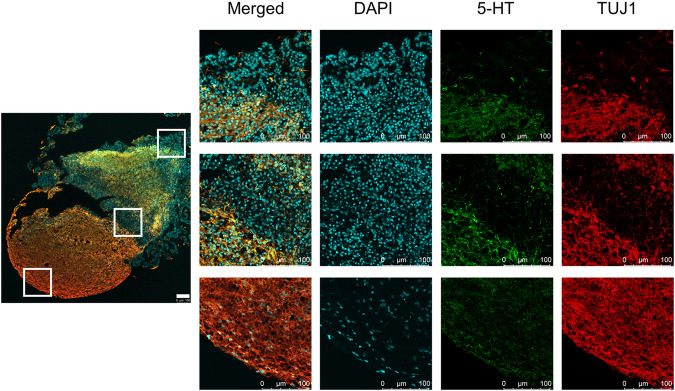
Immunocytochemistry for the 5-HT organoids. Confocal images of the organoids show distinct regions of differentiated cells (scale bar: 100 µm). Neurons are present in most of the organoid area (TUJ1+ cells, in red), and many of them are producing serotonin (stained with 5-HT, green). The overlap between neurons and 5-HT is seen mostly in the middle upper regions (orange/yellow). Regions of the organoid with cells that are not neurons (of any kind) are most prevalent on the edges, where they are also radially arranged in structures reminiscent of the signature architecture of neuronal progenitor cells (NPCs). To confirm this, a second panel of confocal images (Fig. S2) shows the organoid stained with NPC markers Nestin (green) and NKX2.2 (in red).

Confocal images of the organoids confirm the above results and reveal distinct regions of differentiated cells (Fig. 5). In the upper panel of images (Fig. 5a), the presence of differentiated neurons is confirmed in almost all of the organoid area (TUJ1+ cells, in red), many of which are also serotonin-producing (stained with 5-HT, green). The overlap between neurons and 5-HT can be appreciated mostly in the middle upper regions (orange and especially yellow). There are areas in the organoids radially arranged in structures (rosettes) reminiscent of the signature architecture of neuronal progenitor cells (NPCs) in neural tube epithelium. To confirm this, a second panel of confocal images (Figure S2), shows the organoid stained with NPC markers Nestin (green) and NKX2.2 (in red). Indeed, cellular regions of the 5-HT-organoid corresponding to those areas with no TUJ1 and no 5-HT staining are confirmed to be NPC, and vice versa. These findings further validate flow cytometry, ELISA and qRT-PCR characterization efforts of our hindbrain organoids (Fig. 4).

### Treatment with Escitalopram Oxalate

**Fig. 6:**
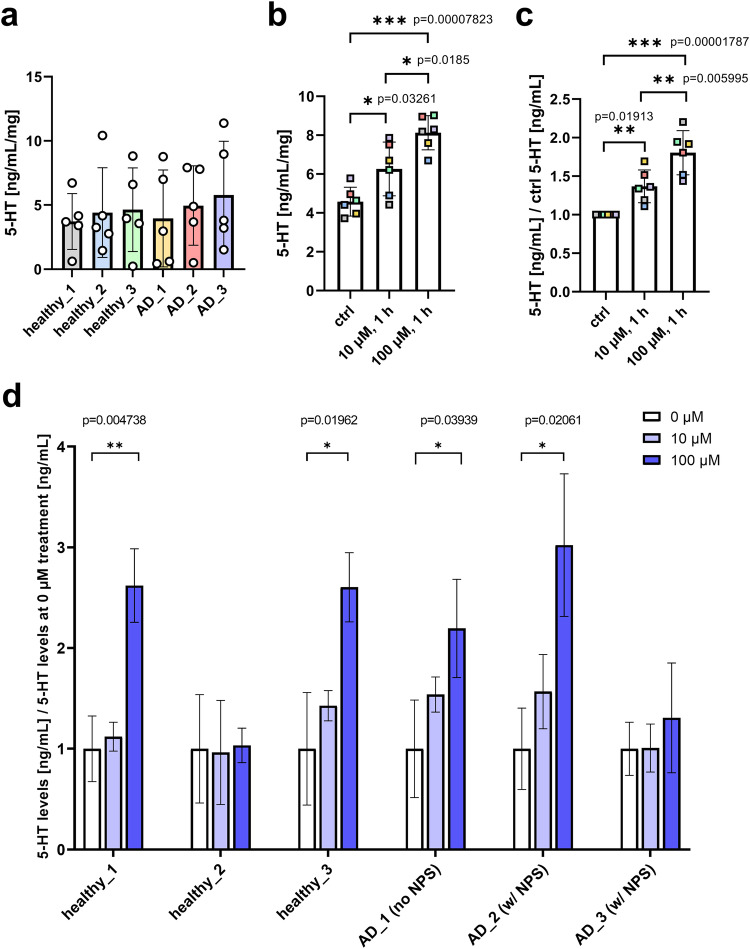
Effect of 1h treatment with escitalopram oxalate on serotonin release from 5-HT-organoids. Baseline serotonin levels at day 42 are similar across all 6 cell lines (**a**) (mean ± SD, *n* = 5). There is a measurable overall effect of 1 h treatment with escitalopram oxalate on the 5-HT organoids when averaging the results from the 6 different individuals (**b**, **c**). The levels of released serotonin are higher upon treatment with the SSRI compared to the baseline control (ctrl), and they are also dose-dependent (mean ± SD, *n* = 6). When looking at the change of serotonin levels individually, healthy_2 and AD_3 do not show any treatment response (**d**) (mean ± SD, *n* = 3).

After establishing an effective in vitro platform for measuring 5-HT release (Fig. 4b), we used it to evaluate the pharmaceutical efficacy of the SSRI escitalopram oxalate on human iPSC-derived 5-HT-organoids. The average baseline levels of serotonin at day 42 for eight 5-HT-organoids were around 4-5 ng/mL, regardless of the individual from whose PBMCs the iPSCs-derived organoids were generated (Fig. 6a). The effect of 1 h treatment with escitalopram oxalate was measurably significant when averaging the results from all individuals, and the response to the drug was dose-dependent when present (Fig. 6b, c). Most interestingly, our 3D in vitro platform indicated that human iPSC-derived hindbrain organoids from different people respond differently to the same dose of drug (Fig. 6d). For individuals healthy_2 and AD_3 there was no observable change in extracellular 5-HT upon treatment with escitalopram oxalate, whereas for the others there was a 2 to 3-fold increase in 5-HT levels. While 1 h of treatment was sufficient to elicit such a response, the higher 100 µM dosage was necessary.

## Discussion

Neuropsychiatric symptoms such as agitation and depression are a prominent feature of AD, and they are strong contributors to the disability affecting patients and their caregivers. Previous studies have shown that early appearance of NPS leads to accelerated cognitive decline in AD [13, 42]. Emerging research suggests a link between NPS in AD and deterioration of the raphe-derived serotonergic system and the serotonin producing neurons therein [43]. At the same time, AD progression, NPS clinical manifestations as well as responsiveness to (few available) pharmacological management options, are all variable, highlighting the increasingly urgent need for a substantially deeper understanding of the underlying pathophysiological mechanisms. This is where we believe our *Precision Medicine* approach of developing clinically relevant, iPSC-derived in vitro models can make a difference.

We report here decidedly robust protocols for the generation of individual iPSC-derived 5-HT-organoids. The hindbrain organoids from human iPSCs offer a viable in vitro platform to study inter-patient variability in AD as part of a *Precision Medicine* strategy. The organoids could be reproducibly differentiated starting from iPSCs, which have been reprogrammed from PBMCs of healthy individuals (*n* = 3) and AD patients clinically presenting with (*n* = 2) or without (*n* = 1) NPS. The organoids’ morphological features and growth over time were quantitatively evaluated over time using the python code we developed for the analysis of BF images. The 5-HT organoids were thoroughly characterized to show the presence of serotonin producing neurons within them by looking into the expression of relevant markers using flow cytometry (TUJ1, TPH2), qRT-PCR (NKX2.2, TPH2, FEV) and ICC (Nestin, NKX2.2, TUJ1, TPH2, 5-HT). The production of 5-HT could successfully be monitored by ELISA for a month after the initial 6 weeks of differentiation. The extensive characterizations with these different assays set the necessary foundation for individualized in vitro testing of the SSRI escitalopram. Two of the six cell lines (healthy_3 and AD_1) gave rise to 5-HT organoids which were not responding to the drug, while the remaining four responded in a dose-dependent manner.

The different responses to escitalopram we measured in hindbrain organoids originating from different individuals suggest that this in vitro method can be used to identify subgroups of AD patients that may be more likely to respond to therapy with escitalopram in vivo. We propose that patient-specific hindbrain organoids can be effectively used for drug screening purposes to predict pharmacological benefits. At a minimum these methods can help us understand the heterogeneity of response to treatment.

Retention of pathological identity would be key to study the pathophysiology of AD using iPSC models. Indeed, this is a problem that cuts across all uses of iPSC models to study specific diseases since it is likely that any epigenetic pathological identity is erased in the reprogramming process [22]. However, it is very unlikely that genetically determined pathological identity is erased. And this extends beyond pathological identity related to a disease that is built into DNA, to genetically determined identity that relates to response to the application of medications such as Escitalopram. The present study examines inter-individual differences in Escitalopram effects on serotonin reuptake that are presumably genetically determined. It utilizes an in vitro model meticulously developed using different cell lines with diverse diagnostic backgrounds, precisely to ensure the platform’s broader applicability. The heterogeneity of organoid responses to SSRI exposure may reflect heterogenous clinical responses which we see in clinical practice. The strategies developed herein should be repeated on a larger scale of iPSC-derived samples and eventually compared to clinical outcomes from the (still ongoing) S-Citad study.

## Supplementary information

Supplemental material

## Data Availability

All generated and analyzed data is included in the article and its supplementary information files. The Python code is available as an open resource on github [38]. Supplementary information (SI) is available at MP’s website.
