# Supplementary material for: iPSC-derived hindbrain organoids to evaluate escitalopram oxalate treatment responses targeting neuropsychiatric symptoms in Alzheimer’s disease
Source: Mol Psychiatry. 2024 Jun 5;29(11):3644–52. doi: 10.1038/s41380-024-02629-y (PMC11541203; doi:10.1038/s41380-024-02629-y)
Supplement: Supplementary file 1 — Supplemental material [file 41380_2024_2629_MOESM1_ESM.docx]

# **iPSC-derived hindbrain organoids to evaluate escitalopram oxalate treatment responses targeting neuropsychiatric symptoms in Alzheimer’s disease – Supplementary Information**

Cristina Zivko, PhD, Ram Sagar, PhD, Ariadni Xydia, B.S., Alejandro Lopez Montes, PhD, Jacobo Mintzer, MD, Paul Rosenberg, MD, David M. Shade, J.D., Anton Porsteinsson, MD, Constantine G. Lyketsos, MD, Vasiliki Mahairaki, PhD^*^

The following document contains supplementary tables (Tables S1-S3) and supplementary figures (Figures S1,S2) from the above-mentioned manuscript.

**Table S1: Medium composition for 5-HT-organoids differentiation.** Serotonergic NPC medium (SNm) is used the first 3 weeks, and neural differentiation medium (NDm) afterwards. Medium was freshly prepared on the day it was used. Components are listed alongside their final concentrations. Unless otherwise stated, all material was purchased from Gibco, PreProtech and Tocris.

**Table S2: List of antibodies used for ICC and flow cytometry.**


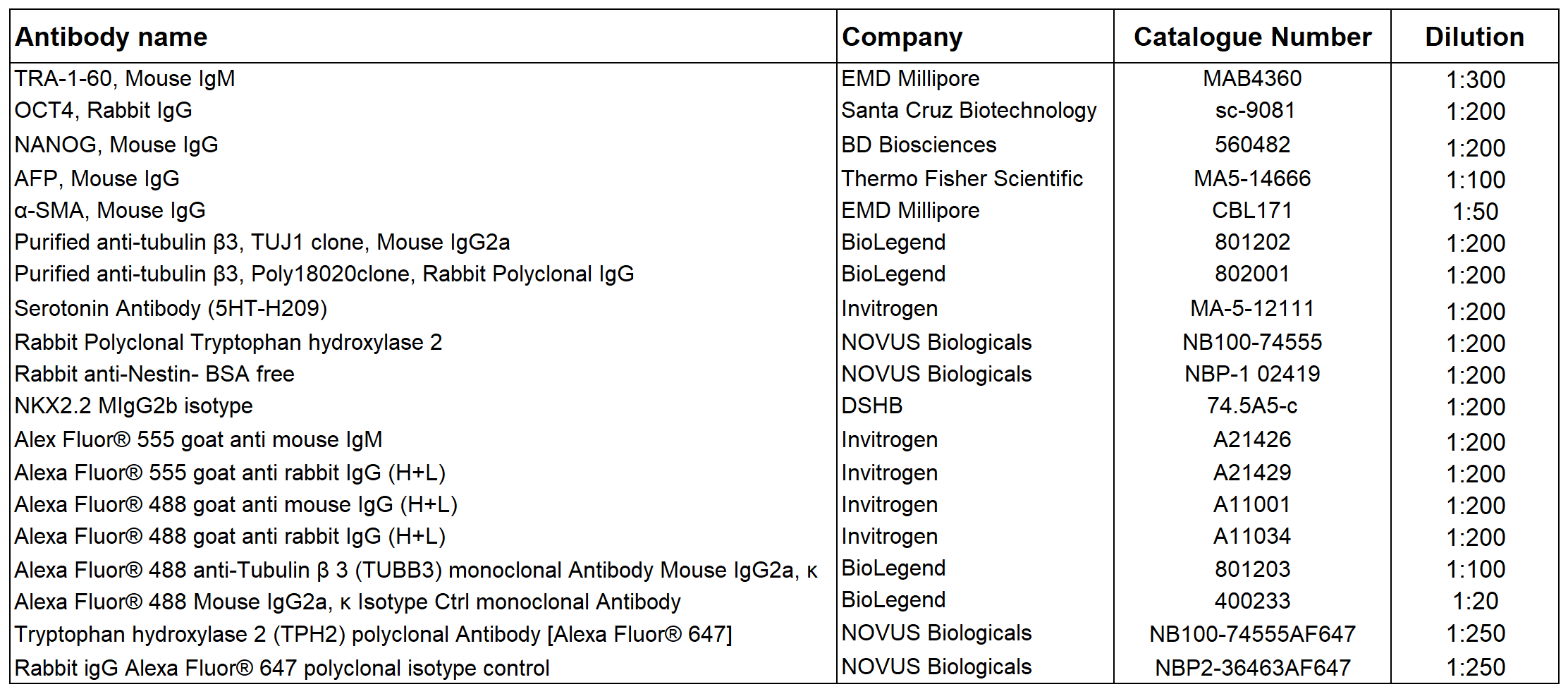


**Table S3**: List and sequences of oligo DNA primers.

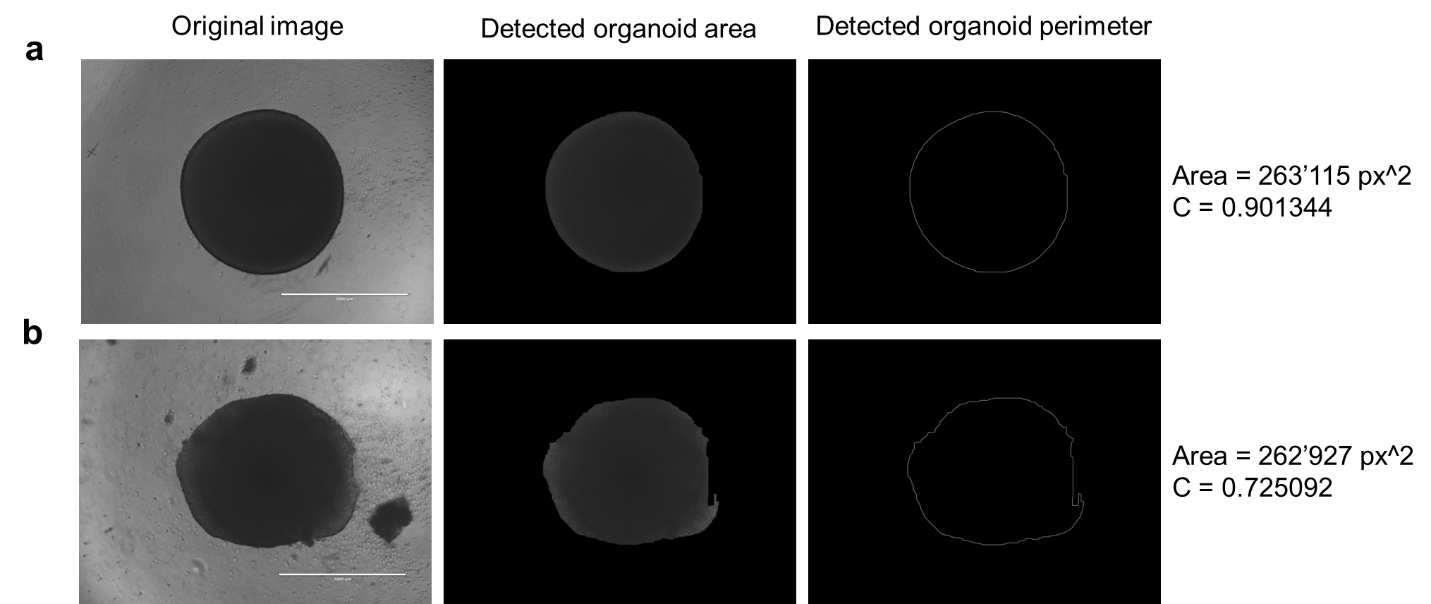


**Figure S1: Representative segmentation results.** Area and perimeter of the organoids can be detected with sufficient precision using our code. The near perfect circularity of some organoids can be quantitatively expressed with C values approaching 1 (**a**), whereas less round perimeters will result in lower C values (**b**). Scale bar: 1000 µm.


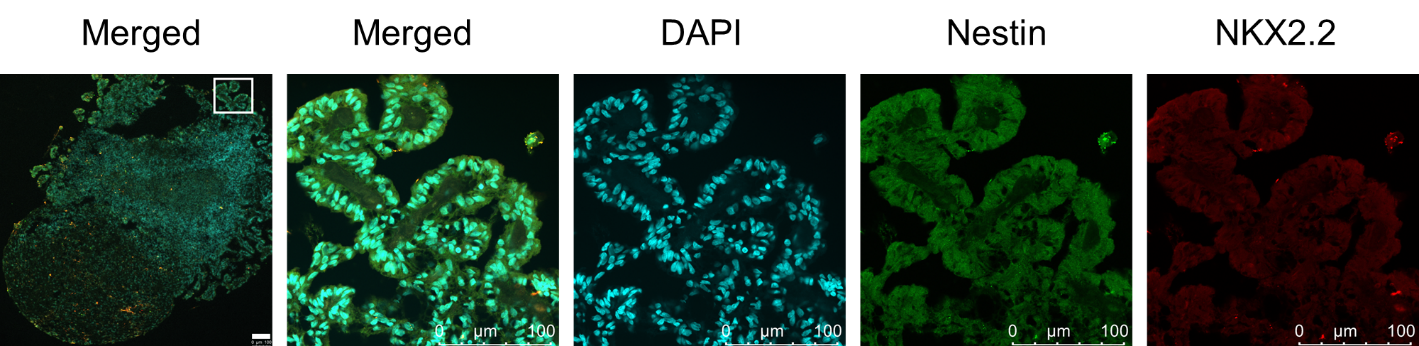


**Figure S2:** Organoid stained with NPC markers Nestin (green) and NKX2.2 (in red). Scale bar: 100 µm.


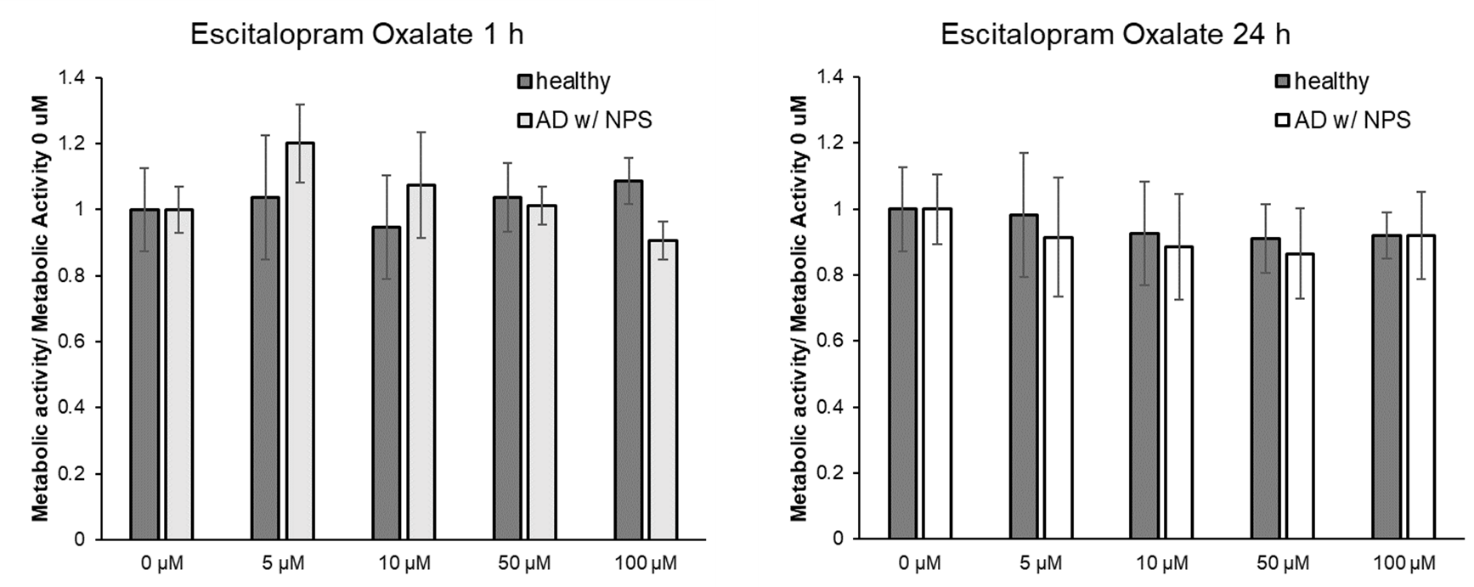


**Figure S3:** A standard metabolic activity assay was performed using CyQUANT^TM^ MTT Cell viability assay kit (invitrogen, Cat. Nr. V13154) according to manufacturer’s instructions after either 1 or 24 h of treatment with different concentrations of Escitalopram Oxalate. There was no measurable difference from the non-treated respective controls in the concentration range used (mean ± SD, n=1, N=6).
